# The Predictive Potential of Altered Voxel-Based Morphometry in Severely Obese Patients With Meibomian Gland Dysfunction

**DOI:** 10.3389/fnins.2022.939268

**Published:** 2022-07-07

**Authors:** Le-Yan Li, Yuan-Yuan Wang, Jun-Wei Gao, Jun Chen, Min Kang, Ping Ying, Xulin Liao, Yixin Wang, Jie Zou, Ting Su, Hong Wei, Yi Shao

**Affiliations:** ^1^Department of Ophthalmology, Jiangxi Branch of National Clinical Research Center for Ocular Disease, The First Affiliated Hospital of Nanchang University, Nanchang, China; ^2^Department of Clinical Medicine, Queen Mary School, Nanchang University, Nanchang, China; ^3^Department of Radiology, The First Affiliated Hospital of Nanchang University, Nanchang, China; ^4^Department of Ophthalmology and Visual Sciences, The Chinese University of Hong Kong, Hong Kong, Hong Kong SAR, China; ^5^School of Optometry and Vision Sciences, College of Biomedical and Life Sciences, Cardiff University, Cardiff, United Kingdom; ^6^Department of Ophthalmology, Massachusetts Eye and Ear, Harvard Medical School, Boston, MA, United States

**Keywords:** meibomian gland dysfunction, obesity, voxel-based morphometry, MRI, predictive potential, cognitive impaiment

## Abstract

**Objective:**

To investigate voxel-based morphometry (VBM) by using magnetic resonance imaging (MRI) in meibomian gland dysfunction patients with severe obesity (PATs) and to explore the application of VBM in the early diagnosis, prevention of cognitive impairment and targeted treatment of this disease.

**Methods:**

Sixteen PATs and 12 healthy controls (HCs) were enrolled and underwent MRI. Whole-head images were analyzed using VBM and data were compared between groups using an independent samples *t*-test. Receiver operating characteristic (ROC) curves were utilized to assess the diagnostic value of this approach. Mini-mental state examination (MMSE) scores were used to assess cognitive impairment and were analyzed using an independent samples *t*-test.

**Results:**

Compared with HCs, the VBM values in PATs were reduced in the left cerebellum and right thalamus but increased in the right brainstem, right precuneus and right paracentral lobule. The results of ROC curve analysis indicated that VBM may be useful in meibomian gland disease diagnosis. Comparison of MMSE scores between groups showed mild cognitive impairment in PATs.

**Conclusion:**

PATs showed altered VBM values in some brain areas. These findings may provide information about the pathophysiology of meibomian gland dysfunction and may help to explain the underlying mechanisms of clinical manifestations in PATs, such as cognitive impairment. Abnormal VBM values in these brain areas may serve as predictive factors for development of meibomian gland disease in severely obese people and as indicators for individualized treatment.

## Introduction

Meibomian gland dysfunction (MGD) is a condition commonly encountered by ophthalmologists which can lead to dry eye (Bron and Tiffany, 2004) and with prevalence of 46 to 70% in Asian populations (Lin et al., 2003; Lekhanont et al., 2006; Uchino et al., 2006). Manifestations include ocular discomfort, increased tear evaporation, decreased visual acuity, and cognitive impairment (Labbé et al., 2013; Chhadva et al., 2017). Typical characteristics of MGD are the blockage of meibomian gland terminal ducts and abnormal glandular secretions, disrupting ocular surface homeostasis (Chhadva et al., 2017). MGD is associated with several genetic and environmental etiologies, including dyslipidemia (Kuriakose and Braich, 2018), which is responsible for the lipid and secretory changes. Dyslipidemia is commonly found in severely obese populations, and the compositions of lipids in the meibomian gland can be changed due to the abnormal state of lipids in the blood, disrupting the protective function (Dao et al., 2010). Destruction of the lipid protection can increase the risk of inflammation and is positively correlated with the incidence of MGD (Kuriakose and Braich, 2018). The body mass index (BMI) prescribed by the World Health Organization is a metric widely used to evaluate obesity (World Health Organization Regional Office for the Eastern Mediterranean, 2010). For adults, a BMI over 30 kg/m^2^ defines severe obesity.

Without gold-standard methods of diagnosis and treatment, a range of costly and complicated but ineffective options are available for application in MGD patients (Thode and Latkany, 2015). Since MGD is closely associated with cognitive impairment (6), an understanding of the pathophysiological changes in MGD is very important for early diagnosis and targeted treatment. Magnetic resonance imaging (MRI) enables researchers to investigate alterations in brain morphology and activity non-invasively (Brown et al., 2016). Medical research has developed a range of MRI analytical techniques, such as functional connectivity (Hutchison et al., 2013), degree centrality (Xiong et al., 2021), fractional anisotropy (Zhou et al., 2021), and fractional amplitude of low-frequency fluctuation (Shao et al., 2015) to identify differences between patients and healthy individuals. In our previous studies, the latter method showed functional brain anomalies in severely obese MGD patients, but brain morphological status in this condition remains unclear.

Voxel-based morphometry (VBM) is another MRI technique and is used to assess structural compositions of individual voxels in whole brain volume, gray matter volume (GMV) and white matter volume (Ashburner and Friston, 2000). In recent years, the VBM approach has been used in ocular diseases such as retinal detachment (Li et al., 2020), proliferative diabetic retinopathy (Xiao et al., 2021), and acute eye pain (Lan et al., 2019). A previous study found that severely obese individuals had increased risk of cognitive impairment such as Alzheimer’s disease, a neurodegenerative disorder caused by reduced GMV (Gustafson et al., 2003). Another study showed that higher BMI values are linked to reduced brain volume (Song et al., 2015).

The VBM technique has been shown to be an accurate and reliable indicator of abnormal brain morphology and underlying pathological mechanisms. However, few VBM studies have focused on severely obese MGD patients (PATs). To the best of our knowledge, this study is the first to investigate voxel-wise differences between PAT and healthy control (HC) groups using the VBM approach.

## Materials and Methods

### Subjects

Sixteen PATs (5 males, 11 females) were enrolled at the First Affiliated Hospital of Nanchang University, Ophthalmology Department. Corneal confocal microscopy was used to identify abnormal meibomian glands. The volunteers met the following criteria: (1) Diagnosis of MGD; (2) BMI ≥ 30 kg/m^2^; (3) no other ocular disease; (4) no history of ocular surgery; (5) no mental illness; (6) no abnormal cerebral infarction; and (7) no drug or alcohol abuse.

Twelve HCs (six males, six females) were also enrolled according to the following inclusion criteria: (1) Age and educational level matched to PATs; (2) 18.5 kg/m^2^ < BMI < 25 kg/m^2^; (3) no mental illness or ocular disease; (4) no history of eye surgery or cerebral injury; and (5) no drug or alcohol abuse. All volunteers in both groups were required to have no MRI scan contraindications.

The Hospital Ethics Committee approved this study. The study protocol was in line with the Declaration of Helsinki.

### Magnetic Resonance Imaging Data

All volunteers were scanned using a 3T MR scanner (Siemens, Munich, Germany) in a quiet environment. Cerebral T1-weighted images were captured using magnetization prepared rapid acquisition with gradient echo (MP-RAGE) with the following settings: 176 image scans; 1.0 mm thickness; 0.5 mm gap; 250 mm × 250 mm field of view; 256 mm × 256 mm acquisition matrix; 9-degree flip angle; 2.26 ms echo time; and 1,900 ms repetition time.

### Image Preprocessing

All data were previewed using MRIcro software to minimize redundant information. Cerebral structural images were pre-processed using MATLAB version 7.9.0 with the VBM toolbox 8 (VBM8) in Statistical Parametric Mapping version 8. VBM8 automatically marked the position of white and gray matter and cerebrospinal fluid according to the international consortium for brain mapping and normalized all images to the DARTEL (Diffeomorphic Anatomical Registration through Exponentiated Lie Algebra) template following the criteria defined by the Montreal Neurological Institute to standardize the locations of white and gray matter. Volumes were smoothed using a 6-mm full-width at half maximum Gaussian kernel to minimize noise and improve image quality. The preprocessed image was presented for the intergroup analysis.

### Data Processing

An independent samples *t*-test was conducted in the REST toolbox to assess the voxel-wise differences in MRI data between the PAT and HC groups using voxel level *P* < 0.001, AlphaSim, and cluster size > 54 voxels for multiple comparison. The mini-mental state examination (MMSE; Tombaugh and McIntyre, 1992) and activity of daily living scale were conducted to measure levels of cognitive impairment and daily functional status, respectively, in PATs and HCs. The SPSS 24.0 software and GraphPad Prism 9 were used to conduct an independent samples *t*-test between groups. Receiver operating characteristic (ROC) curves were used to estimate the diagnostic values of VBM for MGD in severe obesity. *P* < 0.05 was regarded as significant.

## Results

### Demographics and Behavioral Manifestations

**FIGURE 1:**
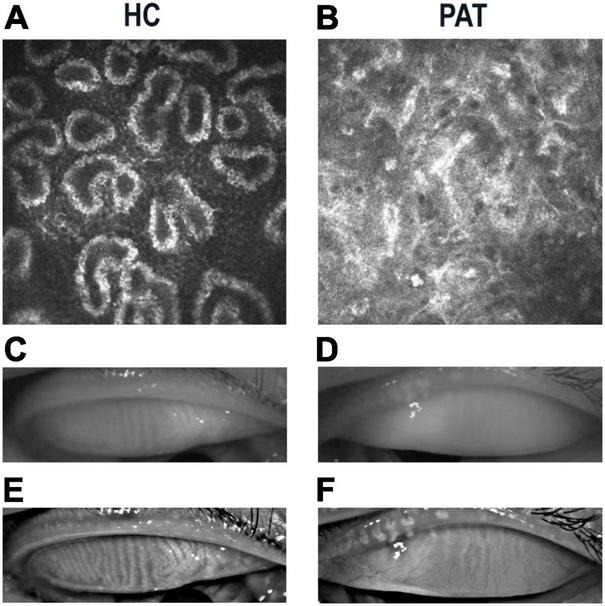
Representative manifestations of meibomian gland in HCs and PATs. Meibomian gland photographed under corneal confocal microscopy in HC **(A)** and PAT **(B)**. Significant blockage of meibomian glands in PAT group. Photograph of meibomian gland as seen by the naked eye in HC **(C)** and PAT **(D)**. Infrared photography of meibomian gland in HC **(E)** and PAT **(F)**. Clearer meibomian gland in HC group. Abbreviations: HC, healthy control; PAT, severely obese patients with meibomian gland disease.

The PAT and HC groups were similar in age (*P* = 0.994) but differed significantly in weight, bilateral initial visual acuity, daily life score and MMSE score (*P* < 0.05; Table 1). Figure 1 shows representative meibomian gland appearance *via* corneal confocal microscopy, the naked eye and infrared photography.

**TABLE 1:** Characteristics of participants in the study.

Condition	PATs	HCs	*t*	*P*-value
Male/female	5/11	6/6	N/A	0.333
Age (years)	31.69 ± 7.69	31.67 ± 5.98	0.008	0.994
Weight (kg)	113.44 ± 14.50	66.08 ± 10.41	9.258	<0.001
Initial visual acuity-left eye	0.78 ± 0.18	0.59 ± 0.09	3.517	<0.01
Initial visual acuity-right eye	0.81 ± 0.21	0.62 ± 0.13	2.926	<0.01
Daily life score	90.50 ± 6.91	100.00 ± 0.00	5.325	<0.001
MMSE score	22.63 ± 4.36	27.83 ± 2.41	3.599	<0.01

*Independent t-test (P < 0.05 significant) comparing two groups. Data are means ± standard deviations.*

*Abbreviations: PATs, severely obese meibomian gland disease patients; HCs, healthy controls; N/A, not applicable; and MMSE, mini-mental state examination.*

### Voxel-Based Morphometry Levels

**FIGURE 2:**
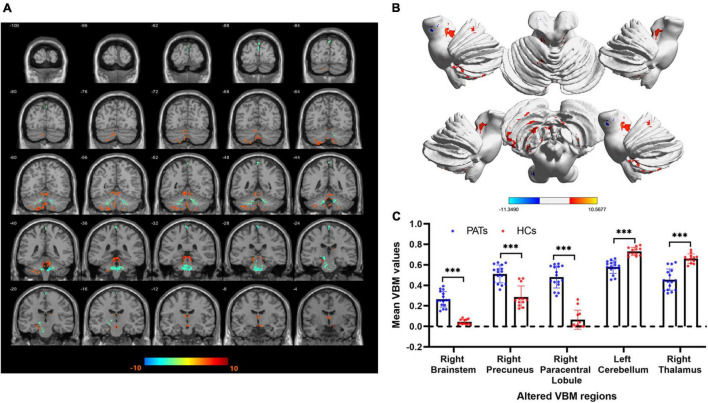
VBM differences between the PATs and HCs. **(A,B)** Areas with lower VBM values are marked in blue, while those with higher VBM values are marked in red. **(C)** Mean VBM values in the brain areas showing differences between PATs and HCs. Abbreviations: VBM, voxel-based morphometry; PATs, severely obese meibomian gland disease patients; and HCs, healthy controls. ***P < 0.001.

Compared with the HC group, VBM values were significantly higher in the PAT group in the right brainstem, right precuneus and right paracentral lobule were significantly higher in PAT than HC, and significantly lower in the left cerebellum and right thalamus (Figure 2 and Table 2). The mean VBM values of each group are shown in Figure 2C.

**TABLE 2:** VBM brain regional differences between the PATs and the HCs.

Condition	Brain areas	MNI coordinates					
		*X*	*Y*	*Z*	BA	Peak voxels	*T* value
**PATs < HCs**							
Left cerebellum		–8	–70	–20		95	7.07
Right thalamus		2	–6	8		105	7.13
**PATs > HCs**							
Right brainstem		4	–34	–38		2,091	–10.88
Right precuneus		2	–88	18	19	56	–5.87
Right paracentral lobule		0	–28	74	4	134	–11.35

*Independent t-tests comparing the two groups. Threshold was set at the voxel level with P < 0.001, AlphaSim, and cluster size > 54 voxels for multiple comparison.*

*Abbreviations: PATs, severely obese patients with meibomian gland disease; HCs, healthy controls; BA, Brodmann area; and MNI, Montreal Neurological Institute.*

### Receiver Operating Characteristic Curve

**FIGURE 3:**
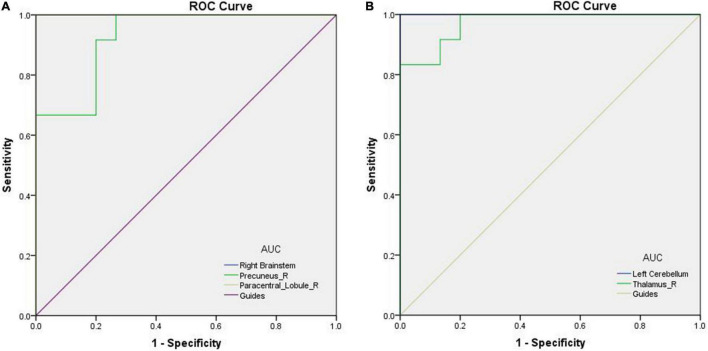
ROC curves of the mean VBM values for abnormal brain areas in PATs. **(A)** AUC of the ROC curve: 1.000 for right brainstem (95% CI 1.000–1.000, *p* < 0.0001); 0.928 for right precuneus (95% CI 0.834–1.000, *p* < 0.0001); 1.000 for right paracentral lobule (95% CI 1.000–1.000, *p* < 0.0001). **(B)** The AUC of the ROC curve: 1.000 for left cerebellum (95% CI 1.000–1.000, *p* < 0.0001); 0.972 for right thalamus (95% CI 0.921–1.000, *p* < 0.0001). Abbreviations: VBM, voxel-based morphometry; AUC, area under the curve; ROC, receiver operating characteristic; and PATs, severely obese patients with meibomian gland disease.

The areas under the ROC curves (AUCs) of VBM in areas with increased values in PATs were as follows: 1.000 in right brainstem (95% CI 1.000–1.000, *p* < 0.0001), 0.928 in right precuneus (95% CI 0.834–1.000, *p* < 0.0001) and 1.000 in right paracentral lobule (95% CI 1.000–1.000, *p* < 0.0001; Figure 3A). The AUCs of VBM in areas with decreased values in PATs were as follows: 1.000 in left cerebellum (95% CI 1.000–1.000, *p* < 0.0001) and 0.972 in right thalamus (95% CI 0.921–1.000, *p* < 0.0001; Figure 3B).

## Discussion

**FIGURE 4:**
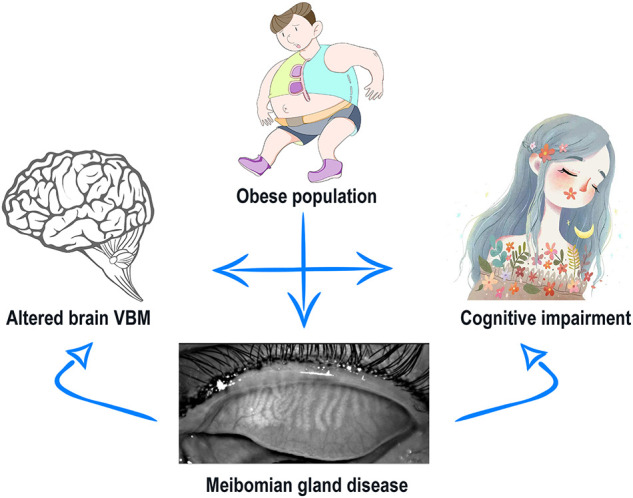
Relationship between altered VBM values and cognitive impairment in severely obese patients with meibomian gland disease. Severely obese have increased likelihood of meibomian gland disease (MGD). MGD then leads to altered VBM values in specific brain areas of PATs compared with HCs. PATs are more likely to have cognitive impairments, which may correlate with the abnormal brain VBM. Abbreviations: VBM, voxel-based morphometry; PATs, severely obese patients with meibomian gland disease; and HCs, healthy controls.

Meibomian gland dysfunction is a common public health concern with increasing prevalence, particularly in severely obese populations (Dao et al., 2010). VBM values in specific brain areas are lower in patients with MGD than in HCs and MGD is always accompanied by cognitive impairment (Labbé et al., 2013; Figure 4). Researchers have investigated the underlying pathophysiological mechanisms using a number of approaches and VBM MRI analysis has already been extensively applied in ophthalmic patients and obese populations and is expected to offer significant opportunities for clinical diagnosis (Table 3).

**TABLE 3:** VBM method applied in ophthalmic patients and obese population.

	Author	Year	Disease	Brain areas	
				PATs > HCs	PATs < HCs
Ophthalmic patients	Huang et al., 2016	2016	Optic neuritis	LIPL, LFG	LAC, RIPL, RSFG, LPG, LIFG, RIPL, LPG, L/RMFG, LMFG
	Zhao et al., 2021	2021	Neovascular glaucoma	–	STG, LIFG, LMFG, LCA/MFG,
	Xiao et al., 2021	2021	Proliferative diabetic retinopathy	BLP	LP, LC, LMCG, LOIFG, LT LMTG, BSTG
	Su et al., 2022	2022	Strabismus and amblyopia	–	LACC, RSTG, BPG, P/ALC
Obese population	van Bloemendaal et al., 2016	2016	Type 2 diabetes	–	RIPL, LEC
	Kotkowski et al., 2019	2019	Metabolic syndrome	–	PC, brainstem, OC, BCN, RP, RA, RI, LG, RSTG
	West et al., 2020	2020	Type 2 diabetes	–	MTG, IFG
	Turan et al., 2021	2021	Eating disorder	R/LMOFC	–

*Abbreviations: VBM, voxel-based morphometry; PAT, patient; HC, healthy control; LIPL, left inferior parietal lobule; LFG, left fusiform gyrus; LAC, left anterior cingulate; RIPL, right inferior parietal lobule; RSFG, right superior frontal gyrus; LPG, left precentral gyrus; LIFG, left inferior frontal gyrus; RIPL, right inferior parietal lobule; LPG, left postcentral gyrus; L/RMFG, left and right middle frontal gyrus; LMFG, left middle frontal gyrus; STG, superior temporal gyrus; LIFG, left inferior frontal gyrus; LMFG, left middle frontal gyrus; LCA/MFG, left cingulum anterior/medial frontal gyrus; BLP, bilateral lenticular putamen; LP, left precuneus; LC, left cerebellum; LMCG, left middle cingulum gyrus; LOIFG, left orbital inferior frontal gyrus; LT, left thalamus; LMTG, left middle temporal gyrus; BSTG, bilateral superior temporal gyrus; LACC, left anterior cingulate cortex; RSTG, right superior temporal gyrus; BPG, bilateral parahippocampal gyrus; P/ALC, posterior and anterior lobes of the cerebellum; RIPL, right inferior parietal lobe; LEC, left external capsule; PC, posterior cerebellum; OC, orbitofrontal cortex; BCN, bilateral caudate nuclei; RP, right parahippocampus; RA, right amygdala; RI, right insula; LG, lingual gyrus; RSTG, right superior temporal gyrus; MTG, middle temporal gyrus; IFG, inferior frontal gyrus; and R/LMOFC, right and left medial orbitofrontal cortex.*

**FIGURE 5:**
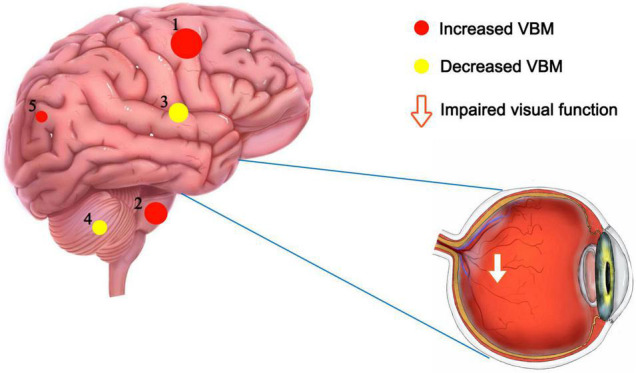
VBM differences between PATs and HCs. Compared with the HCs, the following cerebral regions of PATs showed increased VBM values: 1-right paracentral lobule (*t* = –11.35), 2-right brainstem (*t* = –10.88), 5-right precuneus (*t* = –5.87), and the following regions had decreased VBM values: 3-right thalamus (*t* = 7.13), 4-left cerebellum (*t* = 7.07). Abbreviations: VBM, voxel-based morphometry; PATs, severely obese patients with meibomian gland disease; and HCs, healthy controls.

To the best of our knowledge, this study is the first to assess VBM in severely obese MGD patients. We found that VBM values were significantly decreased in the left cerebellum and right thalamus and increased in the right brainstem, right precuneus and right paracentral lobule (Figure 5).

The cerebellum is a major subcortical area composed of recurrent neural circuit structure. Previous reports have shown that the cerebellum is implicated in motor control (De Zeeuw and Ten Brinke, 2015), emotion (Koziol et al., 2014), and cognitive behavior (Buckner, 2013). Research using VBM approaches have shown significant cerebellar GMV atrophy in patients with mild cognitive impairment (Donzuso et al., 2021) and in those with major depressive disorder (Depping et al., 2020). A previous study found reduced cerebellar degree centrality values in depressed patients with Parkinson’s disease (Wang et al., 2018). Moreover, research on patients with Alzheimer’s disease has shown reduced amplitude of low frequency fluctuation values in the inferior cerebellum compared with healthy individuals (Yang et al., 2018). A previous study also found lower MMSE scores in patients with mild cognitive impairment (Tombaugh and McIntyre, 1992). In line with these findings, we also observed that PATs had lower MMSE scores and markedly decreased VBM in the left cerebellum, indicating brain damage in this region associated with its role in cognition.

The thalamus occupies an important position in the deep brain and contributes to sensory cognition, motor function, as well as emotional processing (Serra et al., 2019). Recent investigations on structural and functional alterations of the thalamus have uncovered their relationships with the severity of cognitive impairment. Some have demonstrated lower thalamic volume in patients with cognitive impairment (Schoonheim et al., 2015). Similarly, thalamus-related white matter volume was found to be degenerated of mild cognitive impairment patients (Zhou et al., 2021). Moreover, patients with mild brain injury and permanent cognitive impairment show reduced fractional anisotropy in the thalamus (Grossman et al., 2012). In the present study, the PATs showed reduced VBM values in the right thalamus, which combined with MMSE results may explain reduced cognitive function in PATs.

The brainstem is an essential part of the central nervous system which consists of the midbrain, medulla oblongata and pons. The brainstem participates in basic human life activities, such as maintaining blood pressure, breathing and controlling self-consciousness (Burdge et al., 2022) and is also responsible for processing sensory information (Russo et al., 2005). Existing studies show that brainstem hypermetabolism leads to cognitive impairment in Parkinson’s disease (Blum et al., 2018). In addition, Ma et al. (2009) showed that significantly increased brainstem activity may be an early feature of Parkinson’s disease. Recent studies have also found that GMV of patients with panic disorder higher in the midbrain and pons compared with controls (Uchida et al., 2008). Results of the present study showed increased VBM in the right brainstem and decreased MMSE scores in PATs, suggesting that meibomian gland dysfunction in severe obesity may lead to cognitive impairment by changing the VBM of the right brainstem.

The precuneus is located in the posteromedial portion of the parietal lobe, and is vital in self-processing and memory retrieval (Cavanna and Trimble, 2006). A previous study demonstrated reduced precuneus activity in the episodic memory encoding process in healthy individuals (Lundstrom et al., 2005). In addition, a positive correlation has been reported between right precuneus neural activation in mental tasks and the incidence of disorganized schizotypy (Acosta et al., 2019). Similarly, increased functional connectivity in the right precuneus has been found in both depressive and non-depressive schizophrenic patients compared to HCs (Li et al., 2021). In the present study, the VBM increase found in the right precuneus of PATs may be related to abnormal cognition.

The paracentral lobule lies medially to the cerebral hemisphere, connects the precentral and postcentral gyri and has a role in cognitive impairment (Mascalchi et al., 2014). A study on patients with mood disorder reported high functional connectivity values in the bilateral paracentral lobule in patients with suicidal behavior (Zhang et al., 2020). Furthermore, previous research on patients with attention deficit hyperactivity disorder revealed increased brain lateralization index in the paracentral lobule when compared with HCs (Zou and Yang, 2021), and increased GMV of the left paracentral lobule in patients with major depressive disorder (Peng et al., 2016). The present study also found increased VBM in the right paracentral lobule, which together with the MMSE score may indicate that meibomian gland dysfunction in severely obese populations affects cognitive information.

The ROC curve analyze was a method used to assess the accuracy of screening potential biomarkers in clinical samples. The AUC > 0.8 is a sign of perfect accuracy. In the present study, all areas of interest fit in perfect AUC values, indicating that the altered VBM could have predictive potential for development of meibomian gland disease in severely obese people.

This study was limited, however, first by small samples, which may influence the accuracy and reliability of the data. Second, the formation of MGD is affected by multiple predisposing factors, which should be controlled in subsequent studies to increase the reliability of results. Third, in the detection of mild cognitive impairment, Montreal cognitive assessment can be superior to the MMSE, which should be replaced in future studies (Pinto et al., 2019).

## Conclusion and Expert Recommendations

The discovery of VBM alterations in five brain areas may provide powerful information about the underlying pathophysiology of severely obese MGD patients. Abnormal VBM values of these brain areas may be reliable, early indicators of MGD onset and development in severe obesity. In addition, cognitive impairment in severely obese MGD patients may be explained by altered VBM values in different brain areas (Table 4).

**TABLE 4:** Brain areas alternations and anticipated results.

Brain areas	Experimental results	Brain function	Anticipated results
Left cerebellum	PATs < HCs	Sensorimotor control, vestibular control, autonomic control, cognitive control, emotional control.	Cognitive impairments, including emotional, attentional, and social problems.
Right thalamus	PATs < HCs	Sensorimotor control, neuropsychological control, cognitive control, emotional control.	Cognitive impairments, including deficits in attention, awareness, memory, and language.
Right brainstem	PATs > HCs	Sensorimotor control, autonomic control, cognitive control.	Cognitive impairments, including deficits in attention and memory.
Right precuneus	PATs > HCs	Sensorimotor control, cognitive control, emotional control	Cognitive impairments, including memorizing and mental disorder.
Right paracentral lobule	PATs > HCs	Part of the default model network	Cognitive impairments, including mental and attentional disorder.

*Abbreviations: HCs, healthy controls; PATs, severely obese meibomian gland disease patients.*

In patients with MGD, the lack of a gold-standard diagnostic method limits therapeutic opportunities. According to current consensus on therapy, the ophthalmologist could only relief patients’ discomfort when the representative symptoms of MGD or even a vision loss are presented. Fully recovery of MGD is hardly observed in patients after treatment. Therefore, medical practitioners should turn the “delayed reaction” therapy to a “prevention and personalized” treatment. Prevention and personalized treatment of the disease would be a better approach than responding to symptoms after its onset. MRI technology may help to optimize prevention, diagnosis and personalized treatment in severely obese MGD patients with more detailed studies in future.

## Data Availability Statement

The datasets presented in this study can be found in online repositories. The names of the repository/repositories and accession number(s) can be found in the article/supplementary material.

## Ethics Statement

The studies involving human participants were reviewed and approved by the Medical Ethics Committee of the First Affiliated Hospital of Nanchang University (Nanchang, China) and followed the principles of the Declaration of Helsinki. The patients/participants provided their written informed consent to participate in this study.

## Author Contributions

L-YL, Y-YW, and J-WG analyzed the data and draft the manuscript. JC, MK, PY, and XL assisted with data interpretation and figure composing. YW, JZ, TS, and HW collected the data. YS conceived, designed and directed the study, and final revised and approved the manuscript. All authors contributed to the article and approved the submitted version.

## Conflict of Interest

The authors declare that the research was conducted in the absence of any commercial or financial relationships that could be construed as a potential conflict of interest.

## Publisher’s Note

All claims expressed in this article are solely those of the authors and do not necessarily represent those of their affiliated organizations, or those of the publisher, the editors and the reviewers. Any product that may be evaluated in this article, or claim that may be made by its manufacturer, is not guaranteed or endorsed by the publisher.
